# From Root Exudates to Eco-Corona: Mechanisms Shaping Nanoplastic Fate and Plant–Soil Interactions

**DOI:** 10.3390/ijms27042080

**Published:** 2026-02-23

**Authors:** Agata Leszczuk, Adrian Zając

**Affiliations:** 1 Institute of Agrophysics, Polish Academy of Sciences, Doświadczalna 4, 20-290 Lublin, Poland; 2 Department of Functional Anatomy and Cytobiology, Institute of Biological Sciences, Maria Curie-Skłodowska University, Akademicka 19, 20-400 Lublin, Poland; adrian.zajac@mail.umcs.pl

**Keywords:** aggregation, eco-corona, nanoplastic, phytotoxicity, plant–nanoplastic interactions, polymer specificity, rhizosphere, root exudates

## Abstract

Plastic contamination in agricultural soils constitutes an emerging threat to plant growth, nutrient acquisition, and food safety. Micro- and nanoplastics (NPs) elicit oxidative stress, perturb root morphology, and interfere with key physiological processes. Despite extensive studies in aquatic systems, the mechanistic understanding of NP behavior in soils, particularly the formation of soil-specific eco-coronas, remains limited. This review provides a mechanistic synthesis of current evidence on the role of root exudates, comprising proteins, amino acids, lipids, and low-molecular-weight metabolites, in modulating NP fate and plant responses within the rhizosphere. We delineate key processes, including exudate adsorption onto NP surfaces, eco-corona formation, aggregation, transport, root uptake, and species- and polymer-specific effects. Root exudation dynamically alters NP surface properties, mediates heteroaggregation, modulates mobility, and regulates interactions with plant roots. At the same time, NP exposure induces species-specific metabolic responses, including enhanced secretion of organic acids, stress-related metabolites, and secondary compounds (e.g., flavonoids). Despite extensive research in aquatic and hydroponic systems, mechanistic understanding of NPs behavior in soils, particularly regarding eco-corona formation and the modulatory role of root exudates, remains limited. This review synthesizes these insights to propose a conceptual framework linking eco-corona dynamics with root exudation processes, thereby providing a foundation for future soil-focused investigations.

## 1. Introduction

Plastic contamination of the environment, including agricultural systems, has become a highly topical and intensively studied research subject. This is unsurprising, considering that the application of plastics in agriculture for regulating field microclimates is projected to increase by approximately 50% by 2030 [1,2,3]. Plastic pollution in soils comprises both microplastics and nanoplastics (NPs), covering the full spectrum of particle types relevant to soil processes and plant interactions. Microplastics are defined as plastic particles with sizes ranging from 1 µm to 5 mm, arising either from the fragmentation of larger plastic materials or intentionally manufactured at this scale. In turn, NPs are plastic particles with dimensions between 1 nm and 1000 nm, typically produced through the further breakdown of microplastics. Available studies detail the complexity of plastic pollution, covering its origins, movement through ecosystems, spatial occurrence, and environmental concentrations [4,5]. While extensive research has characterized NP behavior in aquatic systems, the mechanistic understanding of NP interactions in soils, particularly the formation of soil-specific eco-coronas, remains limited. The rhizosphere, a chemically unique interface enriched with root exudates, plays a central role in mediating NP fate, yet its influence has been largely overlooked.

As the primary interface between plants and soil, roots represent the first point of contact for plastics, influencing their uptake, translocation, accumulation, and phytotoxic effects [6,7]. Root exudates, comprising proteins, amino acids, organic acids, polysaccharides, and secondary metabolites, can adsorb onto NP surfaces, modulate aggregation, and form structured eco-coronas that alter particle mobility, uptake, and toxicity. The accumulation of NPs on root surfaces physically obstructs root pores, thereby limiting water and nutrient uptake and ultimately suppressing plant growth and development [8,9,10,11]. Such alterations in soil physicochemical characteristics and microbial activity also substantially influence plant development [12]. Furthermore, the accumulation of NPs in plant tissues may promote their trophic transfer, thereby posing potential risks to food safety [13,14].

Root uptake is considered the predominant pathway for the entry of micro- and NPs into plant tissues. Beyond particle size, the assimilation of plastic particles by plants is governed by a range of physicochemical characteristics, including particle morphology, concentration, and surface functionalization [14,15,16]. Substantial evidence indicates that NPs penetrate the root epidermis and endodermis through endocytic mechanisms or by translocating across root pores and the cell wall matrix [17,18,19]. Although the presence of Casparian strips restricts the internalization of larger plastic particles, uptake occurs under conditions of root injury, which generate alternative entry points [20,21]. Root surfaces typically possess a net negative charge resulting from the ionization of functional groups such as carboxyl, hydroxyl, amino, and phosphate moieties, within cell walls and plasma membranes [22], facilitating the electrostatic adsorption of positively charged NPs. In contrast, negatively charged NPs, i.e., carboxylated polystyrene (PS) particles, exhibit a greater capacity to traverse the root surface and penetrate internal tissues. In addition to surface charge, particle size represents a critical determinant of NP uptake and translocation. Both nanoscale and submicron plastic particles have been shown to enter plant roots and be transported to aerial tissues, although larger particles tend to accumulate predominantly within root tissues [23]. Ma and co-workers [24] reported that the phytotoxicity of NPs increases as particle size decreases. The mentioned study showed that exposure to small PS nanoparticles (20 nm) markedly suppresses plant growth, reflecting their elevated toxic potential, whereas particles in the 100–1000 nm size range do not cause observable developmental abnormalities. In contrast to larger NPs (≥100 nm), smaller particles exhibit a much greater capacity to injure root cells, particularly within meristematic regions, which are essential for root growth and cellular proliferation. Additionally, the detrimental effects of 20 nm nanoparticles on the meristematic and differentiation zones of *Arabidopsis* roots were found to be concentration dependent. Different plant tissues and organs vary in their sensitivity to nanoscale plastic particles [24].

Following adhesion to the root surface, NPs are translocated toward the central vascular tissues via apoplastic or symplastic pathways. In the apoplastic pathway, NPs are transported with the transpiration stream through the cell wall continuum across cortical parenchyma layers before reaching the endodermis. Next, Casparian strips function as a selective barrier to further apoplastic movement [19]. Conversely, symplastic transport necessitates the passage of NPs through plasmodesmata that interconnect adjacent cells [25,26].

To reduce the stress caused by NPs, plants exhibit various adaptations, ranging from organ to genetic levels [24,26]. At the cellular level, plants activate antioxidant defense systems, with increased production of enzymes such as peroxidases that play a key role in counteracting oxidative stress [27,28]. Ma and co-workers [24] showed that NPs interfere with the formation of cell–cell junctions and disturb pathways related to integral membrane components. These disturbances induce excessive generation of reactive oxygen species (ROS), disrupt cellular turnover, recycling mechanisms, and ultimately result in severe damage to plant cell integrity [24]. Also, Wang and colleagues [21] examined the mechanisms by which PS traverse the external biological barrier of maize roots, revealing that particle-induced deformation of apical epidermal cells and disruption of the protective layer are associated with alterations in cell structure, oxidative stress, and defined pathways of plastic entry [21]. Moreover, exposure to NPs stimulated the biosynthetic pathways of lignin and suberin in plant roots, leading to the formation of a low-permeability barrier that effectively restricted the penetration of NPs [29].

**Figure 1:**
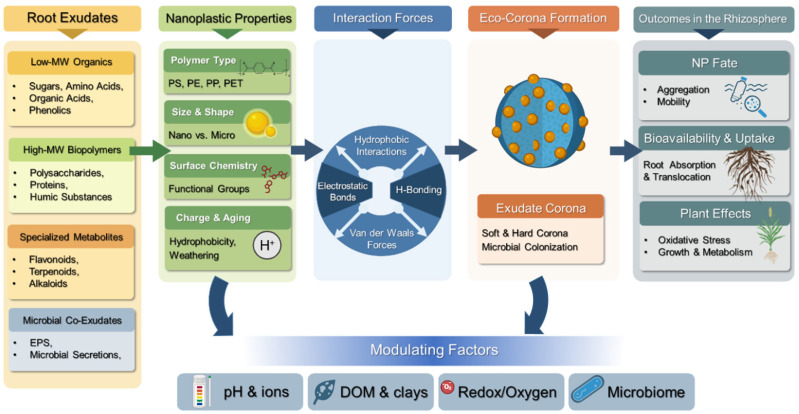
Conceptual diagram illustrating: (i) the composition of root exudates (low-molecular-weight organic compounds, biopolymers, specialized metabolites, and microbial co-exudates); (ii) NPs properties (polymer type, size, surface chemistry, charge, and aging); (iii) dominant interaction forces (hydrophobic interactions, electrostatic forces, hydrogen bonding, and van der Waals forces); (iv) eco-corona formation; and (v) rhizosphere outcomes, including aggregation, mobility, bioavailability, root uptake, and physiological effects on plants, under the influence of soil environmental modulating factors. Created with BioRender.com (accessed on 10 February 2026).

Additionally, plant roots actively secrete a diverse array of root exudates, including low-molecular-weight organic acids, polysaccharide-rich mucilage, and terpenoid compounds, which interact with and bind NPs at the root-soil interface [24,27,30]. Moreover, root exudates significantly facilitated the deposition of micro- and NPs on the root surface, thereby decreasing their mobility within the soil matrix and constraining their subsequent uptake by plants [31]. Despite the strong acceleration of research on NPs, there are still very few reports on whether root anatomy changes in the presence of NPs. Thus, this review addresses the following questions: (i) How do root exudates influence NP surface interactions, eco-corona formation, and aggregation in soil? (ii) What are the mechanistic consequences for NP uptake and translocation? (iii) How do particle properties, plant species, and soil context modulate these processes? The review describes the definition and chemical composition of root exudates, and examines interactions between NPs and root exudates, focusing on exudate adsorption onto NP surfaces and the formation of an ecological corona (Figure 1). By explicitly linking root exudation to NP fate, this review integrates existing mechanistic evidence and proposes a conceptual framework for understanding rhizosphere-mediated NPs dynamics, filling a critical soil-focused knowledge gap that contrasts with the predominantly aquatic-focused literature.

## 2. Effects of NPs on Root Exudation

### 2.1. Chemical Composition and Mechanism of Root Exudate Release

The root exudation phenomenon refers to the process by which plants actively release a variety of organic and inorganic metabolites from their roots into the surrounding rhizosphere. This process is highly dynamic and plays a critical role in mediating plant-environment interactions [32]. During the process, plants release exudates—water-soluble metabolites as well as other plant materials, such as root cap cells, mucilage, plant debris, and volatile compounds. In some crop species, carbon released through root exudation may account for up to 20% of total photosynthetically fixed carbon. However, this value is species-specific as carbon allocation strategies differ substantially between C3 and C4 plants. Root exudates are generally classified into two main groups: low-molecular-weight compounds (LMWCs) and high-molecular-weight compounds (HMWCs) [33]. It is noteworthy that low-molecular-weight compounds encompass a wide range of primary metabolites, such as amino acids, organic acids, and sugars, as well as secondary metabolites, including coumarins, flavonoids, and sorgoleone [34,35]. The most frequently detected amino acids exudates include α-alanine, β-alanine, arginine, asparagine, cysteine, glutamine, glycine, histidine, lysine, methionine, phenylalanine, proline, and serine [36]. The roots predominantly release organic acids into the rhizosphere: citric acid, tartaric acid, and lactic acid. In turn, the most commonly exuded sugars are xylose, fructose, and glucose. Mentioned sugars constitute a major component of rhizosphere mucilage, a viscous matrix that creates a microenvironment for soil microorganisms [35].

Exudate release is mediated through multiple mechanisms, including passive diffusion across cell membranes, facilitated transport via specific carrier proteins, active secretion through plasma membrane transporters, and vesicle-mediated exocytosis [34]. Small and uncharged molecules traverse lipid membranes passively, with the efficiency of this process being influenced by membrane properties and cytosolic pH. Other metabolites, such as amino acids, sugars, and carboxylate anions, are transported in accordance with their electrochemical gradients. The extrusion of protons and the cytosolic potassium gradient facilitate the rapid secretion of carboxylates, while anion channels contribute to their regulation. Two major classes of anion channels have been identified in plant cells: slow-activating anion channels (SLACs), formerly referred to as S-type channels, which require several seconds to become fully activated, and fast-activating anion channels (QUACs), previously known as R-type channels, which respond within milliseconds [33,37]. In turn, the release of high-molecular-weight compounds, including mucilage polysaccharides, primarily relies on vesicular transport. The active release of metabolites from roots is facilitated by specific protein transport systems embedded in the root plasma membrane [33]. Among these systems, two major families of membrane transporters play a central role: the ATP-binding cassette (ABC) transporters and the multidrug and toxic compound extrusion (MATE) transporters [38]. However, the precise mechanisms underlying the secretion of many phytochemicals from root cells remain largely unresolved [39,40,41].

The chemical properties of root exudates govern their mechanistic interactions with NPs. Carboxyl-rich organic acids preferentially bind oxidized NP surfaces through electrostatic interactions, while hydrophobic metabolites associate with nonpolar polymer domains. Polysaccharides and sugars can promote heteroaggregation and reduce particle mobility [33,34,35]. Although experimental data are currently limited, a conceptual categorization of exudates according to their dominant interaction forces, electrostatic, hydrophobic, and hydrogen bonding, provides a mechanistically relevant framework for hypothesizing how root exudation may influence NP aggregation and bioavailability.

### 2.2. The Root Exudates Under Abiotic Stress Conditions

Root exudation is influenced by multiple factors, including plant species and genotype, developmental stage, nutrient availability, soil properties, microbial interactions, and, most prominently, abiotic factors [32]. Different plant species and cultivars exhibit unique root exudation profiles; however, current data do not reveal a clear phylogenetic pattern. Extensive research on root exudation has revealed that the composition and quantity of exudates differ significantly across plant families, species, and even cultivars [42]. Exudation constitutes a highly dynamic process, characterized by temporal variability on a diurnal scale as well as pronounced spatial heterogeneity within the root system. Notably, the root hair zone exhibits markedly elevated rates of metabolite secretion relative to mature root segments. The exudation of total organic carbon peaks during early developmental stages and declines as plants progress into advanced vegetative and later growth stages. Furthermore, during the early stages of plant development, exudation is primarily composed of sugars and organic acids, while the proportion of amino acids becomes more prominent in later developmental stages [32,43].

Abiotic stresses, such as drought, salinity, fluctuations in temperature, soil texture, moisture levels, and nutrient deficiencies, appear to be the primary determinants influencing root exudation, both in terms of quantity and composition [32,43]. For example, under water-stressed conditions, plants often increase the secretion of osmolytes, organic acids, and selected amino acids to maintain adequate rhizosphere hydration and nutrient availability [44]. Salt stress increases the secretion of organic acids, phenolic compounds, and sugars, which support ion chelation, osmotic regulation, and the stimulation of beneficial soil microorganisms [45]. In addition, contaminants, i.e., heavy metals, pesticides, and herbicides, influence root exudation both directly and indirectly [46]. For example, aluminum stress in *Arabidopsis thaliana* enhances the rapid exudation of citrate and malate [47]. Similarly, cadmium exposure modifies root exudate profiles in plants, such as *Sedum alfredii* and *S. plumbizincicola*, involving metabolites like trehalose, organic acids, amino acids, and lipids that either stabilize or mobilize Cd at the soil-root interface [48]. Heavy metals, including Cd, Pb, and Cu, also induce the secretion of phenolic acids, flavonoids, and other secondary metabolites, which contribute to metal immobilization, tolerance, and modulation of rhizosphere microbial activity [49]. While metal-induced exudation is relatively well-characterized, the effects of particulate NPs remain largely unexplored. Unlike ionic toxicants, NPs exert stress through surface-mediated interactions, physical obstruction, and oxidative stress, which may provoke exudation responses distinct from those induced by metals.

### 2.3. Interactions Between NPs and Root Exudates

**Figure 2:**
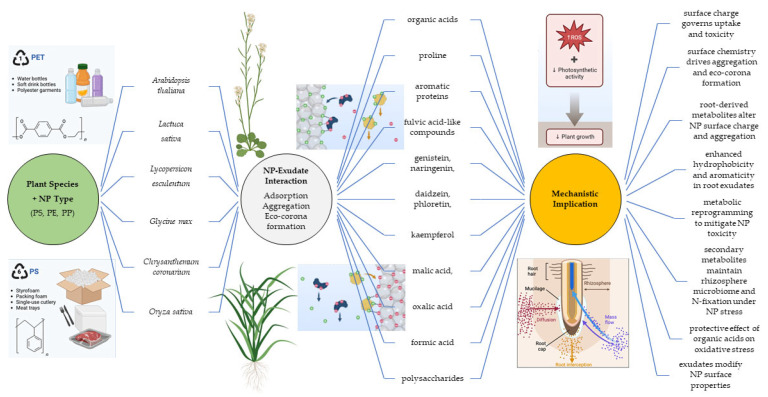
Schematic representation of plant root exudate—NPs interactions and consequences for the plant. Created with BioRender.com (accessed on 10 February 2026).

Current knowledge regarding the influence of root exudates on NP behavior in the rhizosphere remains limited (Table 1). Nevertheless, available evidence indicates that root-secreted compounds can strongly modulate NP spatial distribution (Figure 2) [50].

In the study carried out by Cai [27], the effects of NPs on the chemical composition of root exudates were investigated. In the mentioned research, the authors used polyethylene (PE) with a particle size of 100 µm to investigate the effects of prolonged exposure on lettuce growth. The observed increase in SUVA (Specific Ultraviolet Absorbance) values—SUVA254, SUVA260, and SUVA280—indicates that PE addition led to enhanced aromaticity of root exudates, a greater proportion of hydrophobic and structurally complex organic compounds, and potential qualitative changes in exudate composition as part of the plant’s response to stress induced by microplastics. PE addition increased the SUVA254, SUVA260, and SUVA280 values of root exudates, with the most pronounced effect seen in the 0.05% PE treatment. Moreover, Fluorescence Regional Integration (FRI) analysis of 3D Excitation–Emission Matrix (3D-EEM) spectra showed that root exudates were primarily composed of aromatic proteins (regions I and II), whereas the addition of 0.1% PE led to a slight increase in fulvic acid-like compounds (region III) [27].

Shi and co-workers [30] investigated the phytotoxicity of PS, PE, and polypropylene (PP) in tomato (*Lycopersicon esculentum* L.). Their findings indicated that the effects of microplastics on plant growth were polymer type-dependent, likely reflecting differences in polymer backbones or associated chemical additives. Exposure to microplastics was found to inhibit tomato growth and induce pronounced oxidative stress. Shi [30] evaluated the impact of microplastics on the physio-biochemical traits, root exudation profile, and metabolomic landscape of tomato under hydroponic culture. Following microplastic exposure, the levels of low-molecular-weight organic acids in root exudates were significantly elevated, suggesting a potential adaptive mechanism to mitigate microplastic-induced toxicity. Furthermore, microplastic treatment markedly altered the metabolite composition in both roots and leaves. Pathway enrichment analysis revealed that microplastic exposure substantially affected amino acid metabolism [30].

Furthermore, Shoaib et al. [51] investigated the role of plant root exudates in modulating soil microbial communities and symbiotic performance under exposure to NPs. Their findings indicate that even low-dose NP exposure alters both the quantity and chemical composition of root exudates, thereby affecting the structure and activity of rhizosphere microbiota. Exposure to PP and PE altered the metabolic deposition in the rhizosphere, which reflects changes in exudation patterns. Specifically, soybean plants exposed to NPs exhibited upregulated biosynthesis of flavonoids and isoflavonoids (i.e., genistein, naringenin). These results suggest that under NP stress, plants strategically allocate metabolic resources to enhance the production of these secondary metabolites, thereby safeguarding symbiotic interactions and nitrogen fixation, even at the cost of reduced above-ground biomass accumulation [51]. Similarly, in a 2024 study, Xiao and colleagues [52] examined the response of *Chrysanthemum coronarium* L. to NPs exposure and the modulatory role of root exudates in this context. The authors identified malic acid, oxalic acid, and formic acid as key components of the exudates, collectively accounting for 65.1% of the observed variability in root biochemical parameters and biomass. These organic acids were shown to mitigate oxidative stress by gradually reducing ROS levels in the roots, thereby influencing photosynthetic efficiency and overall plant growth. The study concluded that root exudates, particularly their specific composition of low-molecular-weight organic acids, serve a protective function in plant responses to environmental stressors [52].

The available literature distinguishes three related and mechanistically distinct processes: (i) aggregation, referring to particle–particle association leading to the formation of larger colloidal structures; (ii) molecular adsorption, understood as the surface binding of individual biomolecules to NPs; and (iii) eco-corona formation, denoting the development of an organized, dynamic biomolecular layer on the particle surface.

#### 2.3.1. Aggregation

The low-molecular-weight organic acids, particularly oxalate, released by *Arabidopsis thaliana* roots have been shown to enhance the aggregation of positively charged NPs in a concentration-dependent manner, while exerting minimal effects on negatively charged particles [18]. Aggregation of NPs induced by root-derived exudates significantly reduced the uptake of amino-functionalized PS carrying a positive surface charge. As a result, positively charged NPs accumulated at relatively low levels in root tips; however, despite their limited accumulation, they triggered elevated ROS production and caused stronger inhibition of plant growth than negatively charged sulfonated NPs. In contrast, NPs with a negative surface charge were frequently detected within the apoplastic spaces and the xylem. These findings provide clear evidence that the accumulation patterns of NPs in plants are strongly governed by their surface charge characteristics [18]. Generally, available observations collectively demonstrate that NP size, surface charge, soil characteristics, and root exudation jointly govern the mobility, distribution, and bioavailability of NPs in the rhizosphere [23].

Xu and co-workers [53] demonstrated that exposure to NPs induces an upregulation of organic metabolic processes in roots, leading to enhanced root exudation. The analysis indicated that the clustering positions of essential elements in roots were significantly separated from the control. NPs markedly decreased the levels of key mineral nutrients (K, Ca, Mg, and Fe) in roots, while root exudates facilitated NP aggregation through hydrophobic interactions and bridging mechanisms [53,54]. Root-secreted organic compounds, such as carboxylic acids and amino acids, therefore serve as a key defense strategy against NP-induced toxicity. In Xu’s study [53], NPs exposure led to a significant increase in the secretion of up to 14 organic acids (i.e., hydroxydecanoic acid, glycolic acid, L-aspartic acid). Also, comparative analysis of root exudates revealed that NPs exposure upregulated the TCA cycle and arginine–proline metabolism, promoting the secretion of organic acids and stress-related compounds (e.g., proline), which likely support plant tolerance to NPs-induced stress. These exudates facilitate the heteroaggregation of NPs in the rhizosphere, representing a potential plant self-defense mechanism [53,54].

#### 2.3.2. Molecular Adsorption

Interestingly, in the study conducted by Gu and co-workers [55], the effects of rice root exudates bound to quartz sand surfaces were investigated to assess their influence on the transport of both PS and polyethylene terephthalate (PET) microplastics in porous media. It was found that root exudates from rice roots adsorb onto the surface of quartz sand within porous substrates, thereby reducing the mobility of PS and PET. The observed decrease in microplastic transport was primarily attributed to alterations in the surface properties of the sand induced by the adsorption of root exudate components, rather than the mere presence of plants. The adsorption of these compounds modifies the zeta potentials and electrostatic interactions between microplastics and sand, which, according to the DLVO theory, reduces the electrostatic repulsion and hinders the transport of particles through the porous medium [55].

However, an increasing number of studies report that the interaction between NPs and root exudates is not governed by a single, uniform mechanism; rather, it is NP type-dependent. Notably, the abundant organic acids present in root exudates adsorb onto the surface of nanoparticles, potentially shifting their surface charge to negative [56]. Earlier research has shown that after 6 days of exposure to soybean root exudates, the surface charges of MoO_3_, Cu(OH)_2_, Mn_3_O_4_, and CeO_2_ nanoparticles shifted to a negative state. The presence of organic acids from plant root exudates influenced how nanoparticles aggregated or dissolved. Nanoparticles exhibited different aggregation or disaggregation behaviors depending on the type of nanomaterial and the surrounding environment: i.e., Cu(OH)_2_ and MoO_3_ nanoparticles showed increased aggregate size in root exudates, whereas CeO_2_ and Mn_3_O_4_ nanoparticles tended to disaggregate. Thereby, the presence of root exudates may promote the desorption of other contaminants initially adsorbed onto NPs, thus modifying their phytotoxicity [56].

On the other hand, another study suggests that occurring micro- and NPs with a negative surface charge are more dispersed, and root exudates have no effect on their aggregation. These conclusions are based on research into the environmental weathering of plastics, which leads to the formation of NPs, causing the enrichment of carbonyl groups on the surface of plastic particles [57]. This indicates the possibility of a net negative charge on the surface of NPs isolated directly from the environment. Conversely, negatively charged particles exhibit reduced electrostatic attraction to cell membranes, potentially limiting their cellular uptake. Irrespective of the intrinsic properties of the polymer, the composition of root exudates largely governs the absorption of micro- and NPs and underlies the differential physiological responses observed among plants (Table 1).

**Table 1:** Key Plant Responses and Mechanisms to Nano- and Microplastics Mediated by Root Exudates.

Plant Species	NP Type and Size	NPs—Exudate Interaction	Key Metabolites Identified	Mechanistic Implication	Ref.
**Accumulation Patterns of NPs in Plants Are Strongly Governed by Their Surface Charge Characteristics**					
*Arabidopsis thaliana*	PS (~100 nm)	○ Positively charged NPs showed ~30–40% lower root accumulation than negatively charged NPs; ○ Reduced the uptake of amino-functionalized PS; ○ ROS production was 2-fold higher with positively charged NPs compared with negatively charged	Not specified	Surface charge governs uptake and toxicity	[18]
**Root-Secreted Organic Compounds, Such as Carboxylic Acids and Amino Acids, Serve** **as a Key Defense Strategy Against NPs Toxicity**					
*Lactuca* *sativa*	PE, PS (~200 nm)	○ NP exposure caused a ~20–35% increase in root exudate secretion relative to control; ○ Upregulation of organic metabolic processes; ○ Reduced the contents of key mineral elements in exudates; ○ Exudate-mediated heteroaggregation increased by ~25–50% relative to NP alone	organic acids proline	Surface chemistry drives aggregation and eco-corona formation	[53]
**Root Exudates Adsorb Onto the Surface of NPs, Thereby Modifying Their Phytotoxicity**					
*Glycine max*	NPs (below 1 µm)	○ Root exudates promote the desorption of other; ○ Organic acids from exudates induced an average ~30–60% shift in NP zeta potential toward negative values; ○ Contaminants initially adsorbed onto nanoparticles; ○ Aggregate sizes increased by ~25–45%	Organic acids	Root-derived metabolites alter NP surface charge and aggregation	[56]
**Qualitative Changes in Exudate Composition as Part of the Plant’s Response**					
*Lactuca* *sativa*	PE (~100 µm)	○ PE modified the amount and characteristics of exudates, including a reduction in the concentration of dissolved organic carbon (DOC); ○ DOC in exudates decreased by ~30–40% under 0.1% PE treatments relative to control; ○ PE exposure increased SUVA254 by ~15–25% (indicator of aromatic/hydrophobic compounds); ○ Greater proportion of hydrophobic and structurally complex organic compounds	Aromatic proteins and fulvic acid-like compounds	enhanced hydrophobicity and aromaticity in root exudates	[27]
**Effects of Microplastics on Plant Growth Are Polymer Type-Dependent**					
*Lycopersicon* *esculentum*	PS, PE, PP (<5 mm and <0.1 μm)	○ Significantly elevated levels of low-molecular-weight organic acids in root exudates; increased by ~20–50% after MP exposure vs. control; ○ Microplastic exposure substantially affected amino acid metabolism, showing ~1.5 to 2 fold upregulation in response to MPs	Organic acids, amino acid	Metabolic reprogramming to mitigate NP toxicity	[30]
**Under NPs Stress, Plants Strategically Allocate Metabolic Resources to Enhance the Production of Secondary Metabolites**					
*Glycine max*	PE, PP (~20–50 nm)	○ Low-dose NP exposure alters the quantity and chemical composition of root exudates; ○ Upregulated biosynthesis of flavonoids and isoflavonoids, levels increased by ~35–60% in NP-treated plants	Genistein, naringenin, daidzein, phloretin, kaempferol	Secondary metabolites maintain rhizosphere microbiome and N-fixation under NP stress	[51]
**Specific Composition of Low-Molecular-Weight Organic Acids of Root Exudates**					
***Chrysanthemum coronarium***	PS (~100 nm)	○ Modulatory role of root exudates ○ Malic, oxalic, and formic acids collectively accounted for ~65.1% of observed variability in root biochemical responses under NP; ○ Organic acids were shown to mitigate oxidative stress by gradually reducing ROS levels by ~20–30%	Malic acid, oxalic acid, formic acid	Protective effect of organic acids on oxidative stress	[52]
**Root Exudates Reduce the Mobility of PS and PET Microplastics**					
*Oryza sativa*	PS (~0.51 μm, 1.1 μm), PET (~1 μm)	○ Root exudates adsorb onto the surface of quartz sand within porous substrates; ○ Decrease in microplastic transport—exudate-coated quartz sand reduced the transport of PS and PET by ~40–70% relative to unmodified sand under identical ionic conditions	Low-molecular-weight organic acids, amino acids, polysaccharides	Exudates modify NP surface properties	[55]

### 2.4. Formation of an “Ecological Corona” as a Basic Mechanism of Interaction Between NPs and Exudates

**Figure 3:**
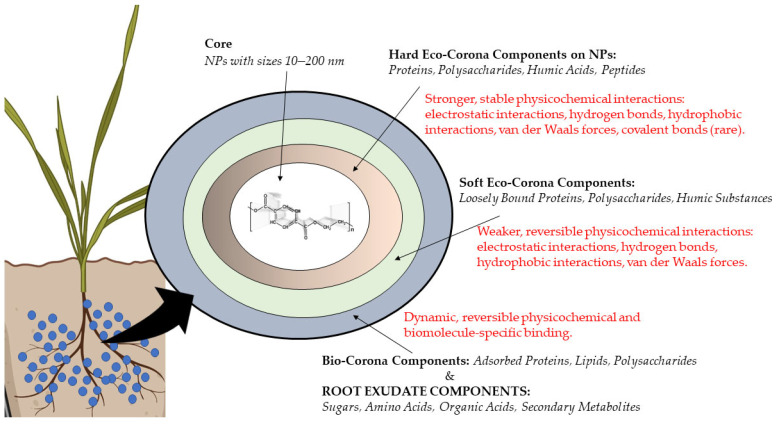
Structural components and interaction forces in eco-corona: hard corona, soft corona, and bio-corona formation on NPs. Created with BioRender.com (accessed on 10 February 2026).

The root exudates contain various proteins and amino acids, which can form a so-called “protein corona” and/or ‘eco-corona’ on the NP surface [58,59,60]. In summary, the eco-corona of NPs in the rhizosphere constitutes a biologically derived layer composed predominantly of root-exuded metabolites, altering the surface properties of NPs and modulating their interactions with plants and the surrounding soil matrix (Figure 3). Biomolecules that are initially present in high abundance can adsorb to the surface of NPs but may later be replaced by molecules exhibiting stronger affinity for the nanomaterial surface [61]. Such dynamic evolution of the eco-corona can occur due to the uptake and relocation of nanomaterials to environments through cellular secretions in response to NPs exposure, which can modify the corona’s composition [62]. These molecules, with molecular weights ranging from 10 to 2,000,000 Da, can adsorb onto nanomaterial surfaces and form a surrounding layer. In some cases, molecules do not bind directly to the nanomaterial surface but instead associate with other biomolecules already present in the eco-corona [63]. Initially, the more abundant proteins attach to nanomaterial surfaces, and they may be displaced by proteins of lower abundance but higher binding affinity [64,65].

The most recent review published by Yang and co-workers [66] explored how the eco-corona modulates the biological behavior of micro- and NPs, emphasizing natural organic matter (NOM) and extracellular polymeric substances (EPS) as representative ecological macromolecules. It discussed the physicochemical characteristics of micro- and NPs, systematically summarized their interactions with macromolecules, and highlighted how eco-corona formation affects particle aggregation [66]. Debroy [67] and Naseer [65] conducted studies on interactions with a diverse array of biomolecules within the aquatic organism, leading to the formation of a ‘biocorona’. Naseer and co-workers [65] primarily focus on how biomolecules present in the aquatic environment (i.e., proteins, polysaccharides, and natural organic matter) adsorb onto the surface of NPs, forming an eco-corona that alters interactions with *Daphnia magna* [65]. The deposition of nanomaterials into environmental waters results in the adsorption of an ecological corona, where a layer of naturally occurring biomolecules coats the nanomaterial, thereby altering its stability [65]. In both cases, the eco-corona altered the surface characteristics of particles, and as a result, substantially affected their aggregation behavior, environmental transport, spatial distribution, and overall toxicity [65,67].

One of the first studies to investigate the influence of root exudation and soil metabolomes on the formation of eco-coronas on plastic surfaces was conducted by Yao and co-workers [68]. The authors proposed that while the eco-corona may not change the inherent sorption capacity of microplastics, it could nonetheless impact how substances adsorb onto their surfaces, for instance, by competing for binding sites and/or modifying the surface characteristics of NPs [68]. The authors found evidence indicating that water-soluble intracellular and extracellular metabolites contribute to the formation of the eco-corona and are actively adsorbed onto NPs surfaces. It was revealed that the concentrations of all-trans-retinoic acid, thymine, aminocaproic acid, betaine, L-isoleucine, L-glutamic acid, phytosphingosine, and deoxyadenosine were significantly reduced in the water-extractable soil metabolites. This indicates that these metabolites were adsorbed onto NPs, contributing to the formation of the eco-corona. Furthermore, it was shown that soil metabolites with high hydrophobicity readily associate with PE microplastics, leading to the formation of an eco-corona. Also, the authors demonstrated that the development of the eco-corona on microplastics in the presence of soil metabolomes occurs rapidly and proceeds spontaneously, as well as persisting in a relatively stable state on the surfaces of the materials for periods ranging from several days to weeks [68].

The binding of biomolecules onto nanomaterial surfaces is governed by a combination of physicochemical forces, including electrostatic interactions, hydrophobic effects, hydrogen bonding, cation bridging, π–π and π–cation interactions, Van der Waals forces, hydrophilic-hydrophilic and hydrophobic-hydrophilic interactions, all of which collectively determine the structure, stability, and dynamics of the resulting interfacial layer [63]. While polymer-dependent differences in plant responses have been observed, the underlying mechanisms remain insufficiently explored, and no specific mechanistic pathways have yet been clearly attributed to individual polymer types. Certain polymers, such as PE or PS, may elicit stronger stress responses due to variations in polymer backbone chemistry, surface properties, and associated additives, including plasticizers and stabilizers [50,51,53,57,58,63].

The formation of an eco-corona on NPs in the presence of soil metabolomes appears to be a widespread phenomenon, altering the surface morphology, functional groups, etc. [68]. While studies on eco-corona formation in aquatic environments have been actively pursued, investigations within soil environments remain largely unexplored. It has not been elucidated which specific biomolecules in root exudates are capable of coating the ecological macromolecules adsorbed on the surfaces of micro- and NPs, thereby establishing a novel eco-corona. In particular, in situ extraction of eco-coronas and kinetic characterization under rhizosphere conditions remain largely unexplored, limiting the direct applicability of models developed in aquatic systems. Soil presents a markedly more complex and heterogeneous matrix, characterized by porous media confinement, spatial variability, and dynamic microbial communities, all of which significantly influence NPs mobility, aggregation, and eco-corona formation [68]. Furthermore, root exudation in soil is often pulsatile rather than continuous, and microbial activities modify the composition and stability of the biomolecular layer. These factors, coupled with challenges in in situ extraction of eco-coronas and kinetic characterization under rhizosphere conditions, underscore the need for soil-specific investigations [68]. Addressing these gaps provides a more accurate framework for understanding NPs behavior and highlights directions for future research in soil-based systems.

## 3. Conclusions

Root exudates have been found to immobilize NPs in the soil, with retention mechanisms showing distinct species-specific patterns. Although existing studies indicate that NP exposure alters plant metabolism and defense responses across various species, the dynamic, time-resolved changes in root exudate composition under such exposure are still poorly characterized. However, in summary, the existing confirmed findings can be highlighted as follows:

1. Definition of root exudates: Root exudates are a chemically diverse mixture of low- and high-molecular-weight compounds, including sugars, amino acids, organic acids, phenolics, proteins, peptides, lipids, and secondary metabolites, secreted by plant roots into the rhizosphere.

2. Determinants of NP effects: The chemical properties of NPs (surface charge, polymer type) together with the composition of root exudates determine nanoparticle uptake and the resulting physiological responses in plants. Positively charged NPs accumulate less in roots than negatively charged ones, induce greater ROS production, and inhibit plant growth more strongly. In contrast, negatively charged NPs more readily penetrate the apoplast and xylem.

3. NP-induced metabolic responses: Exposure to NPs stimulates root organic metabolism, including the TCA cycle, arginine–proline metabolism, and the production of organic acids and stress-related compounds (e.g., proline), potentially acting as a plant defense mechanism against oxidative stress.

4. Three mechanisms of NPs-exudates interaction: (i) aggregation, referring to particle–particle association leading to the formation of larger structures; (ii) molecular adsorption, understood as the surface binding of individual biomolecules to NPs; and (iii) eco-corona formation, denoting the development of an organized, dynamic biomolecular layer on the particle surface.

5. Type of NPs interactions: The formation of the eco-corona is mediated by multiple chemical forces, including electrostatic interactions, hydrophobic effects, and hydrogen bonding.

6. Eco-corona composition and dynamics: Root exudates can adsorb onto NPs surfaces, forming a biologically derived eco-corona that dynamically evolves as higher-affinity molecules replace initially abundant biomolecules. Eco-corona formation alters the surface properties of NPs, influencing their aggregation, stability, transport, spatial distribution, bioavailability, and toxicity.

7. Knowledge gap in soil systems: While eco-corona formation is well-studied in aquatic environments, the specific biomolecules from root exudates that coat or displace ecological macromolecules on NP surfaces in soil remain largely unknown.

Elucidating the molecular mechanisms governing exudate-NP interactions, as well as the impacts of NPs on root morphology and structural properties, could support the development of plant-based approaches in mitigating NP pollution in terrestrial ecosystems. Therefore, research should be undertaken in which the following hypotheses serve as a foundation: (i) interactions between NPs and root-secreted metabolites promote the formation of an eco-corona; (ii) the composition of this eco-corona is plant- or soil-specific due to the variability of root exudates; and (iii) once formed, the eco-corona can influence the sorption and retention of contaminants, potentially mitigating the effects of NPs.

## Data Availability

The raw data supporting the conclusions of this article will be made available by the authors on request.
